# Mice lacking microRNAs in Pax8-expressing cells develop hypothyroidism and end-stage renal failure

**DOI:** 10.1186/s12867-016-0064-x

**Published:** 2016-04-18

**Authors:** Malte P. Bartram, Elena Amendola, Thomas Benzing, Bernhard Schermer, Gabriella de Vita, Roman-Ulrich Müller

**Affiliations:** Department II of Internal Medicine and Center for Molecular Medicine, University of Cologne, Kerpener Str. 62, 50937 Cologne, Germany; Dipartimento di Biologia e Patologia Cellulare e Molecolare, Università degli Studi di Napoli ‘Federico II’, Naples, Italy; Cologne Excellence Cluster on Cellular Stress Responses in Aging-Associated Diseases, University of Cologne, Cologne, Germany; Systems Biology of Ageing Cologne, University of Cologne, Cologne, Germany

**Keywords:** Dgcr8, Cystic kidney disease, Kidney, MiRNA, Thyroid gland, CAKUT, Hydronephrosis, Pax8, Dicer

## Abstract

**Background:**

Non-coding RNAs have gained increasing attention during the last decade. The first large group of non-coding RNAs to be characterized systematically starting at the beginning of the 21st century were small oligonucleotides—the so-called microRNAs (miRNAs). By now we have learnt that microRNAs are indispensable for most biological processes including organogenesis and maintenance of organ structure and function. The role of microRNAs has been studied extensively in the development of a number of organs, so far most studies focussed on e.g. the heart or the brain whilst the role of microRNAs in the development and maintenance of complex epithelial organs is less well understood. Furthermore most analyses regarding microRNA function in epithelial organs employed conditional knockout mouse models of the RNAse III Dicer to abrogate microRNA biogenesis. However, there is increasing evidence for Dicer to have multiple functions independent from microRNA maturation. Therefore Dicer independent models are needed to gain further insight into the complex biology of miRNA dependent processes.

**Results:**

Here we analyze the contribution of microRNA-dependent transcriptional control in Pax8-expressing epithelial cells. Pax8 is a transcription factor that is crucial to the development of epithelial organs. The miRNA machinery was disrupted by crossing conditional *DiGeorge syndrome critical region 8(Dgcr8) fl/fl* mice to *Pax8Cre* mice. The Dgcr8/Drosha complex processes pri-miRNAs in the nucleus before they are exported as pre-miRNAs for further maturation by Dicer in the cytoplasm. *Dgcr8* *fl/fl; Pax8Cre*+ knockout mice died prematurely, developed massive hypothyroidism and end stage renal disease due to a loss of miRNAs in Pax8 expressing tissue.

**Conclusion:**

*Pax8Cre*-mediated conditional loss of DiGeorge syndrome critical region 8 (Dgcr8), an essential component of the nuclear machinery that is required for microRNA biogenesis, resulted in severe hypothyroidism, massively reduced body weight and ultimately led to renal failure and death of the animals. These data provide further insight into the importance of miRNAs in organ homeostasis using a Dicer independent model.

**Electronic supplementary material:**

The online version of this article (doi:10.1186/s12867-016-0064-x) contains supplementary material, which is available to authorized users.

## Background

The *Pax8* gene encodes for a transcription factor that has been shown to play a pivotal role in the development of epithelial organ structures [1, 2]. Expression of cre-recombinase driven by the promoter of *Pax8* has been shown in both kidney epithelia as well as the thyroid gland. Pax8 itself is a transcription factor important in embryogenesis of the thyroid, Müllerian, and renal/upper urinary tracts. Consequently, a *Pax8Cre* mouse line is the perfect tool to address the importance of microRNAs (miRNAs) in the development and maintenance of two important epithelial organs—thyroid gland and kidney—through targeted ablation of miRNA biogenesis.

Regulation of gene transcriptional networks by small non-coding RNAs (miRNAs) has been shown to play a crucial role in epithelial morphogenesis and the development and physiology of epithelial organs. Hence, it is not surprising that loss of microRNAs through conditional knockout of *Dicer* in the thyroid gland using the *Pax8Cre* mouse line resulted in the loss of thyroidal hormone production and death soon after weaning [3, 4]. However, these studies left some major questions unanswered. First, Dicer is not only involved in miRNA biogenesis, but also plays a role in other cellular processes such as genomic DNA fragmentation during apoptosis, the processing of endogenous siRNA and the detoxification of repeat elements [5–8]. Furthermore Dicer has recently been shown to bind to other RNA classes such as tRNAs, snoRNAs, mRNAs and promoter RNAs [9]. In this context Dicer binding does not necessarily involve cleavage but can also primarily impact on RNA-stability and recruitment of RNA molecules to p-granules. One way to confirm a phenotype observed in *Dicer* knockout animals to be truly dependent on miRNAs is confirmation of the phenotype knocking out a different component of the miRNA biogenesis machinery such as *Dgcr8* [10, 11]. Dgcr8 is the interaction partner of the RNAse 3 Drosha and as such essential to the maturation of most miRNAs. This has been done successfully before, e.g. in studies addressing the role of miRNAs in skin development or podocyte maintenance [12, 13]. Dgcr8 itself has a number of functions independent from miRNA biogenesis as well [14, 15]. However, an equal or at least highly similar phenotype between *Dicer* and *Dgcr8* loss of function models would strongly reinforce the hypothesis of the phenotype to be miRNA-dependent since miRNA biogenesis is the only overlapping function between these two proteins known so far.

As to the kidney the specified anatomy of its epithelial composition adds another layer of complexity. In humans each kidney consists of about one million functional units—so called nephrons—that are made up by several specified segments each composed of a number of specialized epithelial cell types. Filtration of the blood occurs in the glomerulus thereby producing the primary urine. The urine is then processed in the tubulus system that can again be divided into several segments each fulfilling a different subset of functions regarding reabsorption and excretion of fluid and electrolytes. These differences in the properties of the tubulus cells along the nephron explain the distinct phenotypes that were described so far in regard to interference with miRNA processing by deleting *Dicer* conditionally in different segments of the tubulus system ranging from no obvious defects at all to rapid end-stage renal disease. For example, *Dicer* was deleted from the entire developing nephron lineage (*Six2Cre*), proximal tubulus (*PEPCK*-*Cre*), in the distal tubulus, collecting duct and developing ureteric bud (*KspCre*) or Wolffian duct, ureter, collecting duct and ureteric bud (*HoxB7Cre*) [16–21].

## Results

To analyse the role of miRNAs in the development and maintenance of two epithelial organs—thyroid gland and kidney—and to understand to which extent the phenotype observed in *Dicer*-*Pax8Cre* knockout mice is indeed due to a lack of miRNAs we generated *Dgcr8*-*Pax8Cre* knockout mice (afterwards named *Dgcr8* knockout).

**Fig. 1:**
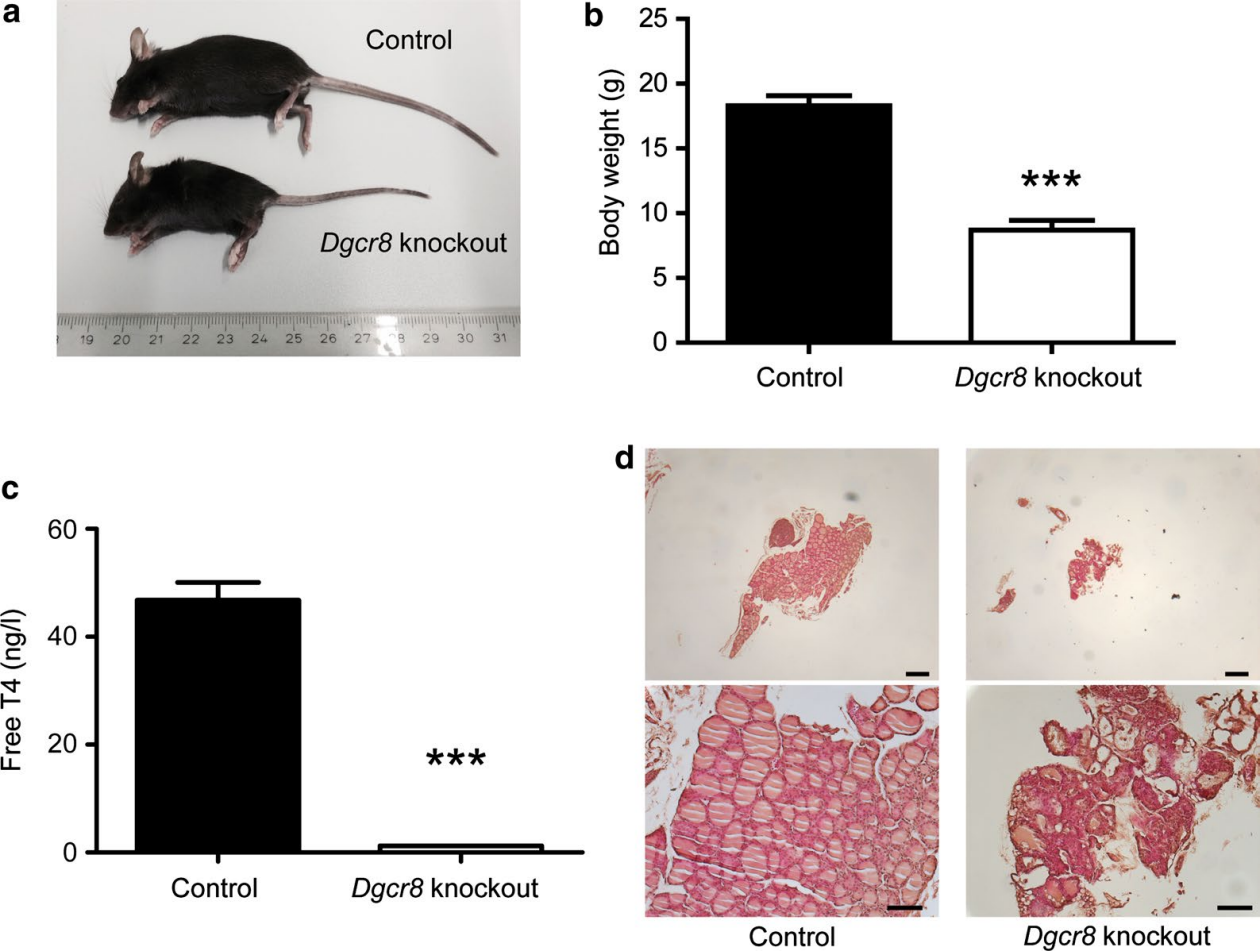
*Dgcr8*–*Pax8Cre* knockout animals develop severe hypothyroidism. **a** and **b** *Dgcr8* knockout mice are smaller and have a reduced body weight (control n = 11; knockout n = 9; age 4–5 weeks; p < 0.0001, *error bars* = SEM). **c** *Dgcr8* knockout mice develop hypothyroidism as demonstrated by dramatically decreased fT4 levels in comparison to control littermates (control n = 5; knockout n = 4; age 4–8 weeks; p < 0.0001, *error bars* = SEM). **d** H&E staining of *Dgcr8* knockout thyroid glands reveals a severely disorganized follicular architecture (*top panel* 5× magnification, *bar* 100 µm; *bottom panel* 20× magnification, *bar* 50 µm)

*Dgcr*8 *fl/fl*, *Pax8Cre*+ offspring was approximately born at the expected mendelian ratio (Additional file 1: Figure S1A). Nonetheless, starting already during the weaning period these mice were smaller and weighing showed a significantly decreased body weight in comparison to littermate controls (Fig. 1 a, b). Analysis of free thyroxine (fT4) revealed massive hypothyroidism in *Dgcr*8 knockout mice since essentially almost no fT4 was detectable anymore (Fig. 1 c). The thyroid glands of *Dgcr8* knockout mice were considerably smaller than the controls. The follicular architecture was almost destroyed, few follicular structures were present with variable diameter and irregular outlines (Fig. 1 d).

**Fig. 2:**
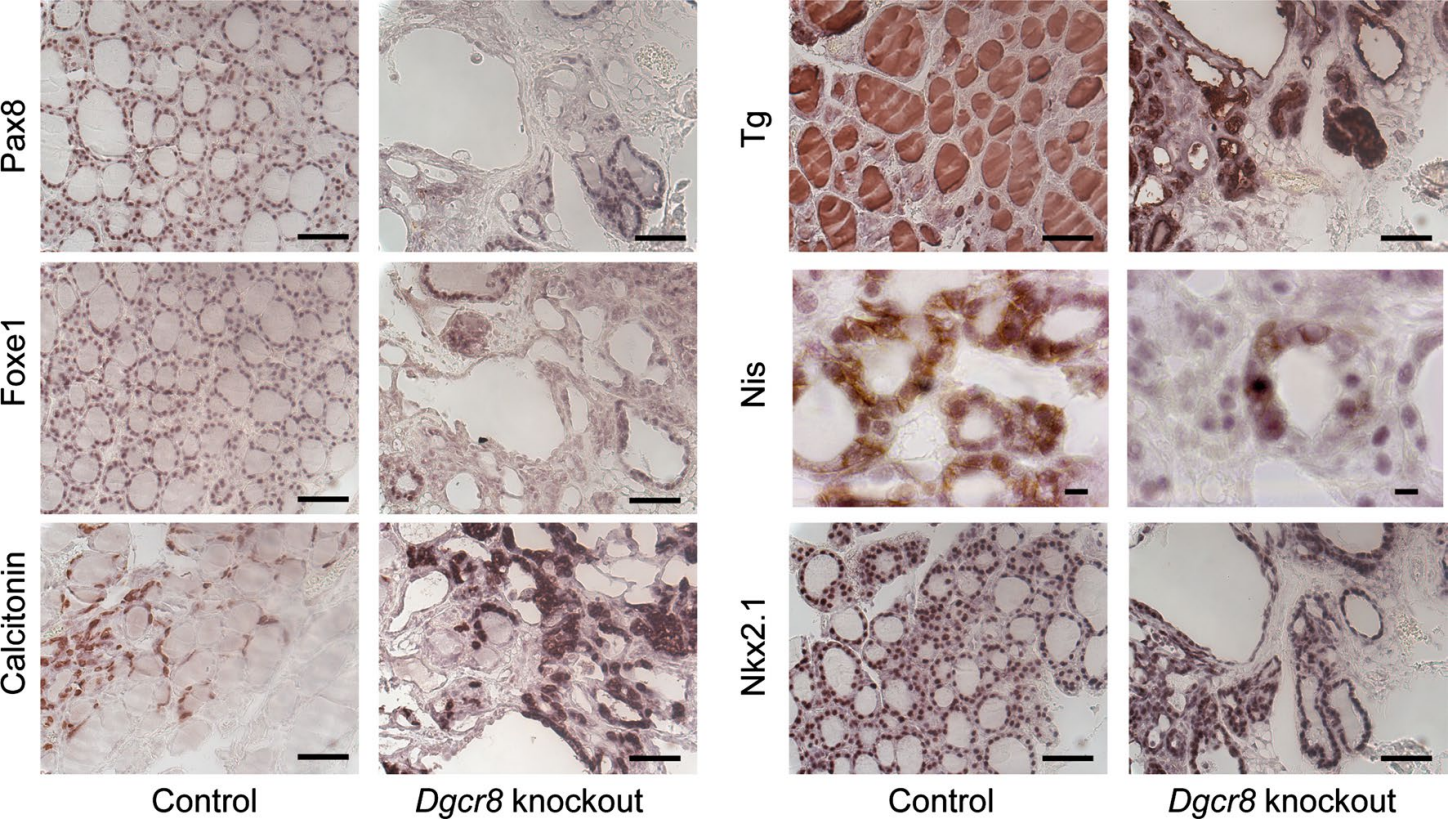
Analysis of differentiation markers in *Dgcr8*–*Pax8Cre* knockout thyroid glands. Immunohistochemistry of thyroid differentiation markers show a strong reduction of Tg expression. Pax8, Nkx2.1 and Foxe1 expression levels are unaffected in the residual follicular cells, as well as the expression of their target Nis. No alteration in calcitonin cells was observed. (40× magnification for Tg, Pax8, calcitonin, Nkx2.1 and Foxe1 *bar* 50 μm; 100× magnification for Nis, *bar* 10 µm, age of the mice 4–8 weeks)

Molecular analysis of differentiation markers on knockout thyroid glands showed that the expression of the early differentiation markers Pax8, Nkx2.1 and Foxe1 is retained in the residual follicular cells, as well as the expression of their target Nis. Conversely, the expression of another late differentiation marker—Tg—resulted strongly reduced in the residual follicles. No alterations in calcitonin secreting cells were observed (Fig. 2). Thus, *Dgcr8* knockout mice show functional defects partly similar to those from *Dicer* knockout mice [3, 4]. The main difference is that Nis is still expressed in *Dgcr8* knockout mice, while being completely absent in *Dicer* knockout animals.

This severe hypothyroidism would generally be enough to explain the lethality of loss of microRNAs in the thyroid gland. However, as mentioned above previous studies showed that death in mice with a *Pax8Cre* mediated loss of *Dicer* in the thyroid gland could not be prevented by thyroxin substitution. This points to an additional mechanism leading to premature death in *Pax8Cre* mediated coditional loss of miRNAs [3].

Beside the thyroid gland it is known that Pax8 is also expressed in the kidney, especially in almost all segments of the kidney tubulus system [22].

**Fig. 3:**
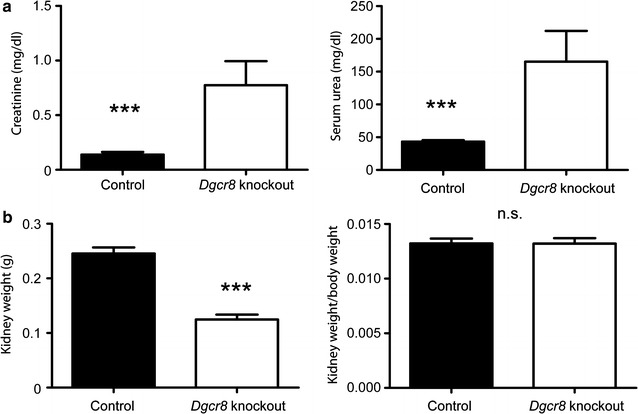
*Dgcr8*–*Pax8Cre* knockout animals develop end stage renal disesase. **a** *Dgcr8* knockout mice develop severe renal failure as shown by increased serum urea (knockout n = 4, control n = 10; p < 0. 0005; *error bars* = SEM) and serum creatinine (knockout n = 4, control n = 10; p < 0. 0009; *error bars* = SEM) levels. **b** The kidneys of *Dgcr8*–*Pax8Cre* knockout mice weigh less than control kidneys (knockout n = 10, control n = 13; p < 0. 0001; *error bars* = SEM) but there is no significant difference in the kidney/body weight ratio between these groups

Kidneys of the *Dgcr*8 knockout mice were smaller and weighed less than the control kidneys but there was no significant difference in the kidney/body weight ratio. Serum urea and serum creatinine levels were substantially increased in *Dgcr*8–*Pax8Cre* knockout mice showing that these mice develop end-stage renal disease at the age of 4–6 weeks (Fig. 3 a, b). Quantitative PCR analysis confirmed the knockout of Dgcr8 in the kidney (Additional file 1: Figure S1B).

**Fig. 4:**
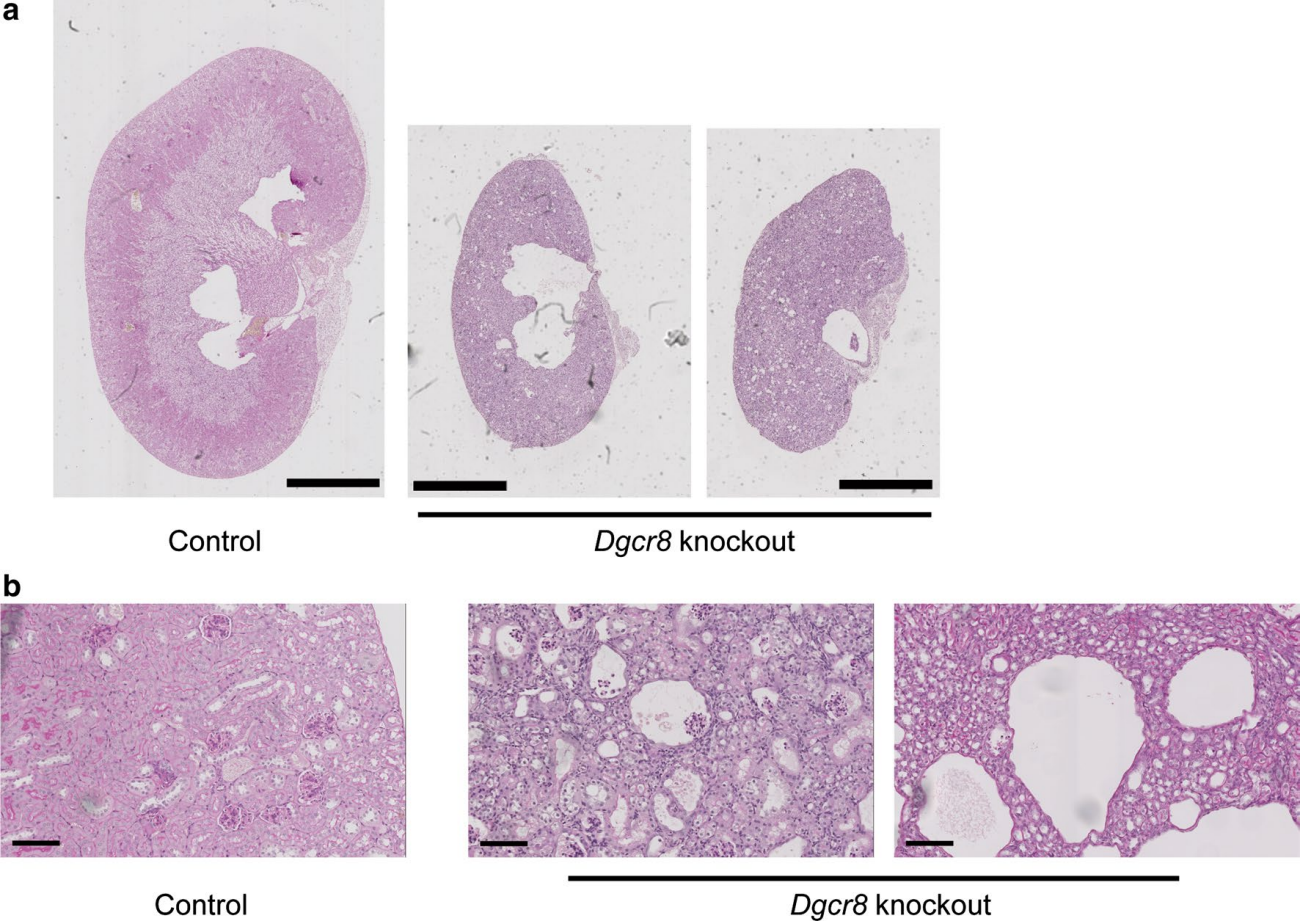
*Dgcr8*–*Pax8Cre* knockout mice have hydronephrotic and cystic kidneys. **a** and **b** PAS staining of the kidneys reveals alterations in all segments of the tubulus system. Beside a general reduction in kidney parenchyma (**a**), there are signs of hydronephrosis, cystic lesions in all parts of the nephron (**b**, glomerulocystic disease *middle panel*, tubular dilations/cysts *right panel*) and a marked activation of the Bowman epithelium (**b**, *middle panel*, see also Additional file 2: Figure S2) (**a** *bar* 2 mm; **b** *ba*r = 100 µm, age of the mice 4–8 weeks)

In line with this finding, histological analysis of the knockout kidneys revealed severe alterations in all parts of the tubulus system. We could detect hydronephrosis, proximal and distal tubular cysts, glomerular cysts and activated Bowman´s epithelium. Also a strongly decreased parenchymal mass was detectable. (Fig. 4 a, b and Additional file 2: Figure S2).

**Fig. 5:**
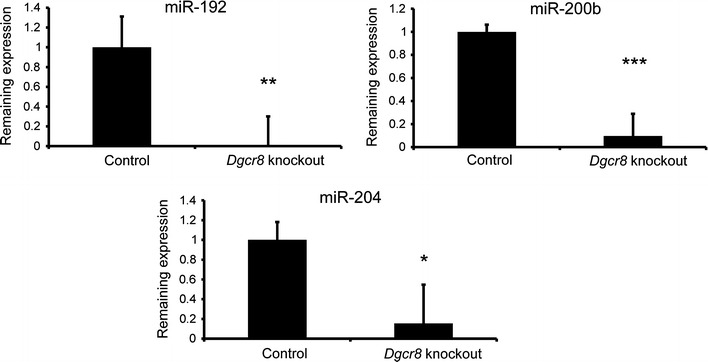
Decreased expression of kidney specific miRNAs. Using qPCR the expression of several miRNAs was analysed. In line with a key role of Dgcr8 in miRNA processing, the kidney epithelial cell enriched miRNAs miR-192, miR-200b and miR-204 are strongly downregulated. SnoRNA-135 served as endogenous control (n = 4 per group, *error bars* = SEM, *p < 0.05, **p < 0.01, ***p < 0.001, age of the mice 4–8 weeks)

Since *Dgcr*8 is one of the key components of efficent miRNA biogenesis we analysed the abundance of several miRNAs that have been reported to be enriched in kidney epithelial cells [16, 17]. The expression of miR-192, miR-200b and miR-204 was significantly decreased in kidneys of *Dgcr*8 knockout mice (Fig. 5).

**Fig. 6:**
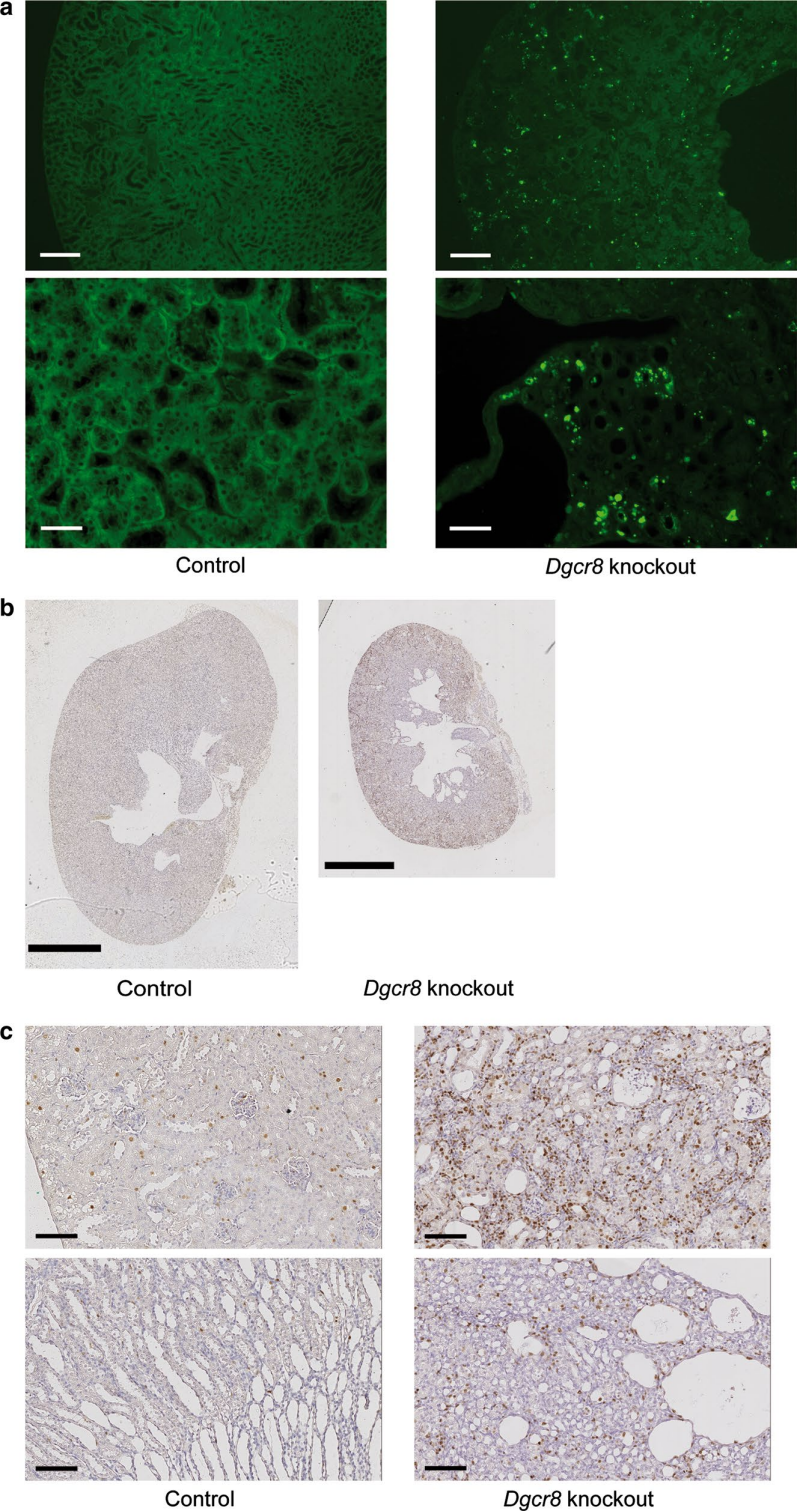
*Dgcr8*–*Pax8Cre* knockout kidneys show increased proliferation and apoptosis. **a** TUNEL staining reveals a massive upregulation of apoptosis in kidneys of *Dgcr8* knockout mice (*upper panel bar* 200 µm; *lower panel bar* 50 µm, age of the mice 4–8 weeks). **b** and **c** An increase in proliferation is detectable especially in the cortex of *Dgcr8* knockout kidneys (**b** *bar* 2 mm; **c** *bar* 100 µm)

To further elucidate the consequences of loss of *Dgcr8* to cellular biology in the kidney, we performed TUNEL assays. These stainings revealed a massive induction of apoptosis in all segments of the kidney (Fig. 6 a), suggesting that *Dgcr8* dependent miRNAs are essential for the development and maintenance of a functional kidney tubulus system. Interestingly staining for the proliferation marker Ki-67 showed that beside apoptosis proliferation is also increased in the tubulus system, especially in the cortical region of *Dgcr*8 knockout mice pointing towards a general increase in cellular turnover (Fig. 6 b, c). Staining of thyroid gland tissue revealed that this increase of apoptosis (determined by TUNEL assays) on the one hand and proliferation (Ki-67 staining) on the other hand is not limited to the kidney but a general finding in the affected epithelial tissue of *Dgcr8*-*Pax8Cre* knockout mice (Additional file 3: Figure S3).

## Discussion

We used the *Pax8Cre*-Allele to induce a conditional knockout of *Dgcr8*. The consequential loss of miRNAs in epithelial cells of the kidney and thyroid gland strongly perturbed the epithelial architecture of these organs leading to massive thyroid hypoplasia and dysplastic kidneys. On the functional side this phenotype is accompanied by not only severe hypothyroidism but also end-stage renal disease leading to premature death of the affected mice. Our mouse model highlights the importance of microRNAs to development and function of two relevant epithelial organs. Just like for most organs the general role of microRNAs in thyroid homeostasis and development had been addressed using a conditional knockout of *Dicer* [3, 4]. Here we show that knockout of *Dgcr8* leads to a similar phenotype of severe hypothyroidism due to thyroid hypoplasia confirming the importance of miRNAs for thyroid development and maintenance. Nonetheless we also observed some structural and molecular differences between *Pax8Cre* mediated loss of *Dicer* and *Dgcr8*. Although the general organization of the thyroid gland of *Dgcr8* knockout mice is severely altered, some structures resembling functional follicles are still present, while *Dicer* knockout mice at the same age do not show any follicular structure. Residual follicles in *Dgcr8* knockout mice show expression of Foxe1 and Pax8 transcription factors, however thyroglobulin is absent from lumina showing instead strong intracellular positivity in some cells. Moreover, *Dgcr8* knockout mice still express the Nis iodide symporter, that resulted absent in *Dicer* knockout thyroids. We can thus speculate that, differently to what observed in Dicer knockout mice, the hypothyroidism observed in *Dgcr8* knockout mice does not depend on a block of differentiation, as the differentiation markers Tg and Nis are expressed in the residual follicles. The inability of thyroid epithelial cells to form functional follicles could be instead due to defects in cell polarization, as suggested by the intracellular retention of Tg.

Whether the differences observed between these mouse models regarding the expression of thyroid differentiation markers are due to specific functions of the small number of microRNAs the processing of which depends only on either Dicer or Dgcr8 or is a consequence of entirely miRNA-independent functions of these proteins remains elusive as of now. Mechanistically seen this will be a highly interesting topic for further research.

The renal phenotype observed provides a good explanation why conditional *Pax8CreDicer* knockout mice could not be rescued by substitution of thyroxin, which would generally rescue the loss of thyroid function [3, 23, 24]. Concomitant end-stage renal failure would inevitably lead to death in this setting. During preparation of this manuscript Iervolino et al. published the renal phenotype of *Pax8Cre* mediated knockout of *Dicer*, which—being largely in line with our findings—again strengthens our view that the phenotype observed is induced by the loss of microRNAs [20]. Renal hypoplasia and hydronephrosis in our mice is in line with the findings observed before regarding *Dicer* knockout using a number of different cre-alleles [16, 18]. Our findings in respect of cell death and proliferation allow for the hypothesis that this perturbed balance is the cell biological correlate of organ dysfunction upon global loss of microRNAs. This cell biological consequence of the loss of miRNAs is not only supported by other studies in different tissues [25–27]. We also found this phenotype in a *KspCre* mediated *Dgcr8* knockout model [28]. As expected taking into account the complex epithelial organization of the kidney described above these mice show a phenotype distinct from the renal disease described here with much more pronounced hydronephrosis.

It will now be an exciting endeavor to clarify whether the dysregulation of cell proliferation and apoptosis is mediated by single miRNA sequences or are rather a consequence of the tremendous effects of a global loss of miRNA-mediated regulatory processes.

## Conclusion

MiRNAs are important regulators of key processes on a cellular, tissue and organismal level. Here we show, that disruption of the miRNA processing machinery using a Dgcr8 dependent knockout in Pax8 expressing cells leads to a severe phenotpye in two epithelial organs that rely on the Pax8 transcription factor during its development. The knockout mice develop severe hypothyroidism due to thyroid gland hypoplasia and develop end stage renal disease with hydronephrosis, cystic kidney disease and hypoplastic kidneys. Consequently this study adds to the evidence of miRNAs being crucial to the normal function of Pax8-expressing epithelial cells. Furthermore the end-stage renal disease observed provides an explanation why thyroid hormone substitution did not rescue mice with a *Pax8Cre* driven conditional knockout of Dicer in previous studies.

## Methods

### Mice

*Dgcr8* fl/fl mice were described previously [11] and provided by Elaine Fuchs (Rockefeller University, NY, USA). The *Pax8Cre* mouse line was a kind gift of Meinrad Busslinger (IMP, Vienna, Austria) [22]. Animals were housed in standardized specific pathogen-free conditions in the animal facility of the CMMC and CECAD (University of Cologne). Animals were fed ad libitum using standard mouse chow. Both female and male mice were used in the experiments. Genotyping was performed according to standard protocols as described previously (Dgcr8 fp: 5´- GACATCAATCTGAGTAGAGACAGG, Dgcr8 rp: 5´- CAGATGGTAACTAACCTGCCAACC, *Dgcr8* wt allel = 244 bp, conditional *Dgcr8* allel = 370 bp; Cre fp: 5´- GCA TAA CCA GTG AAA CAG CAT TGC TG, Cre rp: 5´- GGA CAT GTT CAG GGA TCG CCA GGC G, beta globin fp: 5´- TGC TCA CAC AGG ATA GAG AGG GCA GG, beta globin rp: 5´- GGC TGT CCA AGT GAT TCA GGC CAT CG, Cre allel = 269 bp, beta globin (serves as internal control for the Cre genotyping) = 494 bp). All animal procedures were performed according to European (EU directive 86/609/EEC), national (TierSchG), and institutional guidelines. All procedures as well as the specific study presented here were approved by local governmental authorities (Landesamt für Natur, Umwelt und Verbraucherschutz Nordrhein-Westfalen, LANUV NRW). For the experiments, mice were anesthetized using isoflurane and afterwards killed using the cervical dislocation method.

### Histology

Kidneys of the indicated animals were fixed in formalin, embedded in paraffin and stained with PAS according to standard protocols. To analyse the expression of Ki-67, slides of fixed and paraffin-embedded mouse kidneys were de-paraffinized using Xylol and descending concentrations of ethanol. Antigen retrieval was carried out by warming kidney slides in citrate buffer (10 mM, pH 6) for 10 min using a microwave. After blocking with 3 % H_2_O_2_ and Avidin and Biotin (Vector Laboratories, Burlingame, CA, USA) for 15 min each, slides were sequentially incubated with the Ki-67 antibody (rabbit Ki-67 ab16667, abcam, 1:500 dilution, overnight at 4 °C) and after washing with PBS with biotinylated anti-rabbit IgG (Jackson ImmunoResearch, West Grove, PA, USA; 1 h at room temperature). Kidney slides were labelled with ABC kit (Vector Laboratories, Burlingame, CA, USA), and development was carried out using diaminobenzidine solution (Sigma Aldrich, St. Louis, MO, USA). Slides were counterstained with hematoxylin (Sigma Aldrich, St. Louis, MO, USA), dehydrated and afterwards mounted with Histomount (National Diagnostics).

Stained slides were scanned using a Slidescanner (Leica, Wetzlar, Germany) and analysed using the ImageScope software (version 12.0.1.5030, Aperio).

Thyroids were fixed overnight at 4 °C in 4 % paraformaldehyde in PBS, pH 7.2, dehydrated through ethanol series, cleared in xylene and embedded in paraffin. For histological analysis, 7 µm sections were stained with hematoxylin and eosin (Sigma-Aldrich, St. Louis, MO, USA), according to the manufacturer’s instructions. For immunohistochemical analysis, 7 µm sections were dewaxed by standard techniques (Frezetti et al. 2011). The following primary antibodies were used at the indicated concentrations/dilutions: anti-Pax8 1:500 [29]; anti-Nkx2.1 1:3000 [30]; anti-Foxe1 1:500 [31]; anti-Nis 1:500 [32], anti-Calcitonin rabbit polyclonal antibody 1:2500 (Dako, Milan, Italy), anti-Thyroglobulin rabbit polyclonal antibody 1:500 (Dako), Biotinylated anti-rabbit IgG 1:200 was used for detection of primary antibodies (Vector Laboratories, Burlingame, CA, USA).

### TUNEL assay

To evaluate apoptosis in the kidney we used the Promega DeadEnd Fluorometric Kit according to the manufacturers protocol (Promega, Madison, Wisconsin, USA). Pictures were taken with an inverted microscope (Axiovert200, equipped with an ApoTome system and an AxioCam MRm camera. Objectives used: Plan Apochromat 20 ×/ 0.8 NA and EC Plan Neofluar 5 ×/ 0,16 NA. All from Carl Zeiss, Jena, Germany) using Axiovision 4.8 software for acquisition and subsequent image processing (Carl Zeiss, Jena, Germany).

### QPCR

RNA was extracted from whole mouse kidneys using the phenol–chloroform extraction method. RT reactions were performed using the Taqman microRNA Reverse Transcription Kit (Life Technologies, Carlsbad, California, USA). Expression of miR-192 (assay ID 000491), miR-204 (assay ID 000508) and miR-200b (assay ID 4426961) was analysed with Taqman assays (Life Technologies, Carlsbad, California, USA), snoRNA135 (assay ID 001230) served as endogenous control. To evaluate the expression level of Dgcr8 in the kidney of control and *Dgcr8*–*Pax8Cre* knockout mice, after RNA extraction from the whole kidney the RT reaction was performed using the high-capacity cDNA Kit (Life Technologies, Carlsbad, California, USA). The expression level of Dgcr8 (assay ID Mm.PT.58.33285106, Integrated DNA Technologies, Leuven, Belgium) was normalized against three endogenous controls (Gapdh, assay ID Mm 99999915_g1; Actb, assay ID Mm 02619580_g1; both from Life Technologies, Carlsbad, California, USA; and Hprt, assay ID Mm.PT.58.32092191, Integrated DNA Technologies, Leuven, Belgium). All qPCR experiments were performed on the ABI 7900HT System. Data analysis and statistics were performed using the delta–delta Ct method and a two tailed Student´s t test.

### Laboratory medicine

Blood was obtained by cardiac puncture. Plasma was prepared by centrifugation at 3000 rpm for 10 min. Urea, creatinine and fT4 was measured in the central laboratory medicine unit of the University Hospital of Cologne using the kinetic UV test (urea, Roche Diagnostics), creatinine plus test vers.2 (creatinine, Roche Diagnostics) and fT4 II test (fT4, Roche Diagnostics). Significance was calculated using a two-tailed Student’s t test for all measurements.

## Additional files

10.1186/s12867-016-0064-x *Dgcr8*-*Pax8Cre* mice are born at the Mendelian ratio and reduced expression level of Dgcr8 in *Dgcr8*-*Pax8Cre* knockout kidneys. A *Dgcr8*-*Pax8Cre* knockout mice are approximately born at the expected Mendelian ratio (n = 50 animals). B The expression level of Dgcr8 was examined using qPCR (n = 4 animals per group, error bars = SEM, *** p < 0.001). Since whole kidneys were subjected to the analysis, but conditional knockout is limited to Pax8 expressing cells the remaining expression of Dgcr8 is about 50 % in comparison to the control group.
10.1186/s12867-016-0064-x Additional histological images of *Dgcr8* knockout kidneys. PAS staining of the kidneys of *Dgcr*8 knockout mice shows cysts in the collecting duct, distal and proximal tubulus system, glomerular cysts with activated Bowman epithelium and a reduction in kidney parenchym (bar = 100 µm).
10.1186/s12867-016-0064-x Increased levels of apoptosis and proliferation in thyroid glands of *Dgcr8*-*Pax8Cre* knockout mice. A An increase in apoptotic cells in the thyroid gland of *Dgcr8* knockout animals were detected by TUNEL assays (bar = 100 µm). B Similar to the kidney, there is a marked increase in proliferation as detected by Ki-67 stainings in the thyroid gland of *Dgcr8* knockout mice (bar = 100 µm).

## Abbreviations

CAKUT: congenital anomalies of the kidney and urinary tract
Dgcr8: DiGeorge syndrome critical region 8
miRNA: microRNA
Pax8: paired box 8
Nis: sodium iodide symporter
Tg: thyroglobulin
fT4: free thyroxin
