# Supplementary figures and images for: Mice lacking microRNAs in Pax8-expressing cells develop hypothyroidism and end-stage renal failure
Source: BMC Mol Biol. 2016 Apr 18;17:11. doi: 10.1186/s12867-016-0064-x (PMC4835897; doi:10.1186/s12867-016-0064-x)

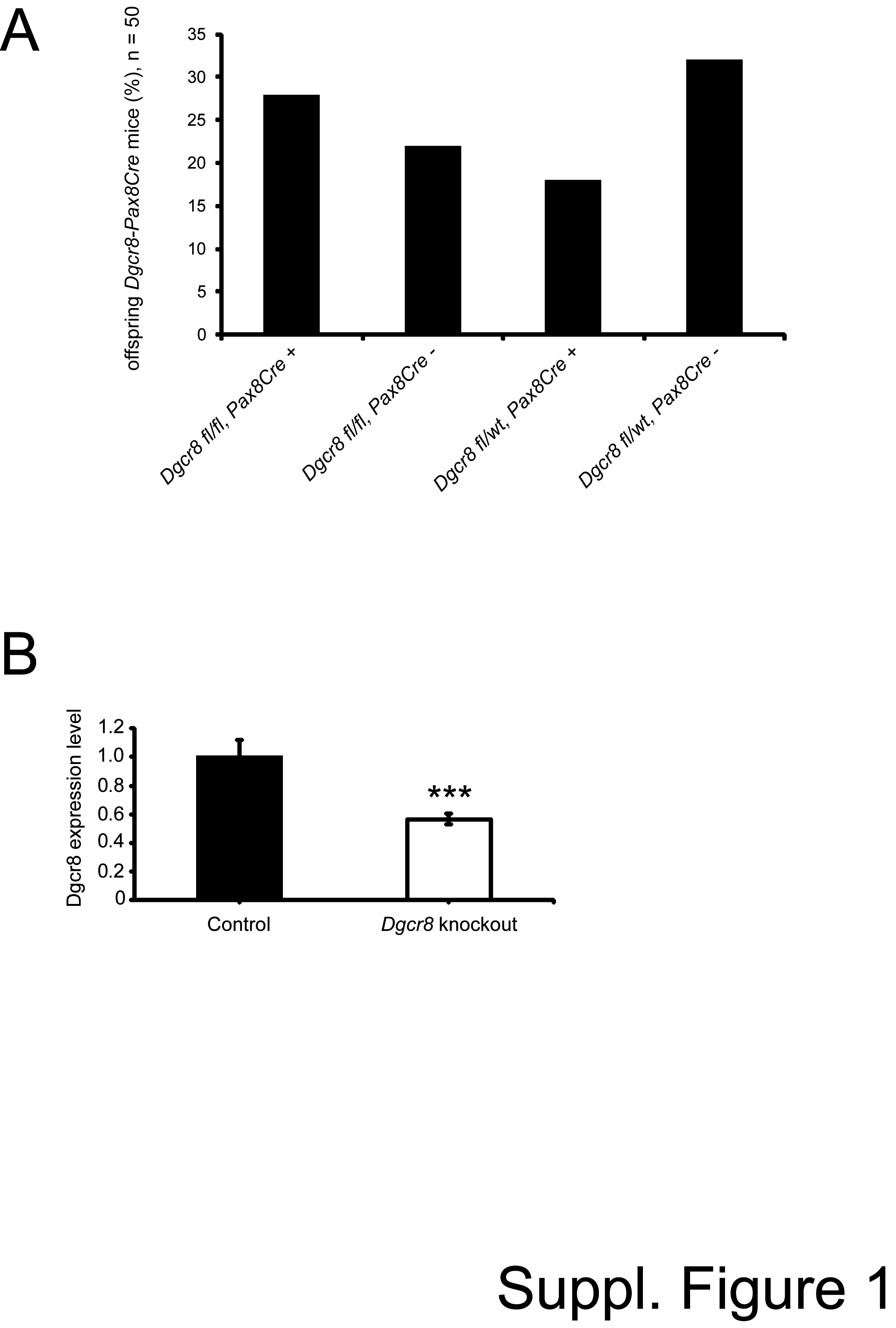

Supplement: Supplementary file 1 — 10.1186/s12867-016-0064-x Dgcr8-Pax8Cre mice are born at the Mendelian ratio and reduced expression level of Dgcr8 in Dgcr8-Pax8Cre knockout kidneys. A Dgcr8-Pax8Cre knockout mice are approximately born at the expected Mendelian ratio (n = 50 animals). B The expression level of Dgcr8 was examined using qPCR (n = 4 animals per group, error bars = SEM, *** p < 0.001). Since whole kidneys were subjected to the analysis, but conditional knockout is limited to Pax8 expressing cells the remaining expression of Dgcr8 is about 50 % in comparison to the control group. [file 12867_2016_64_MOESM1_ESM.tif]

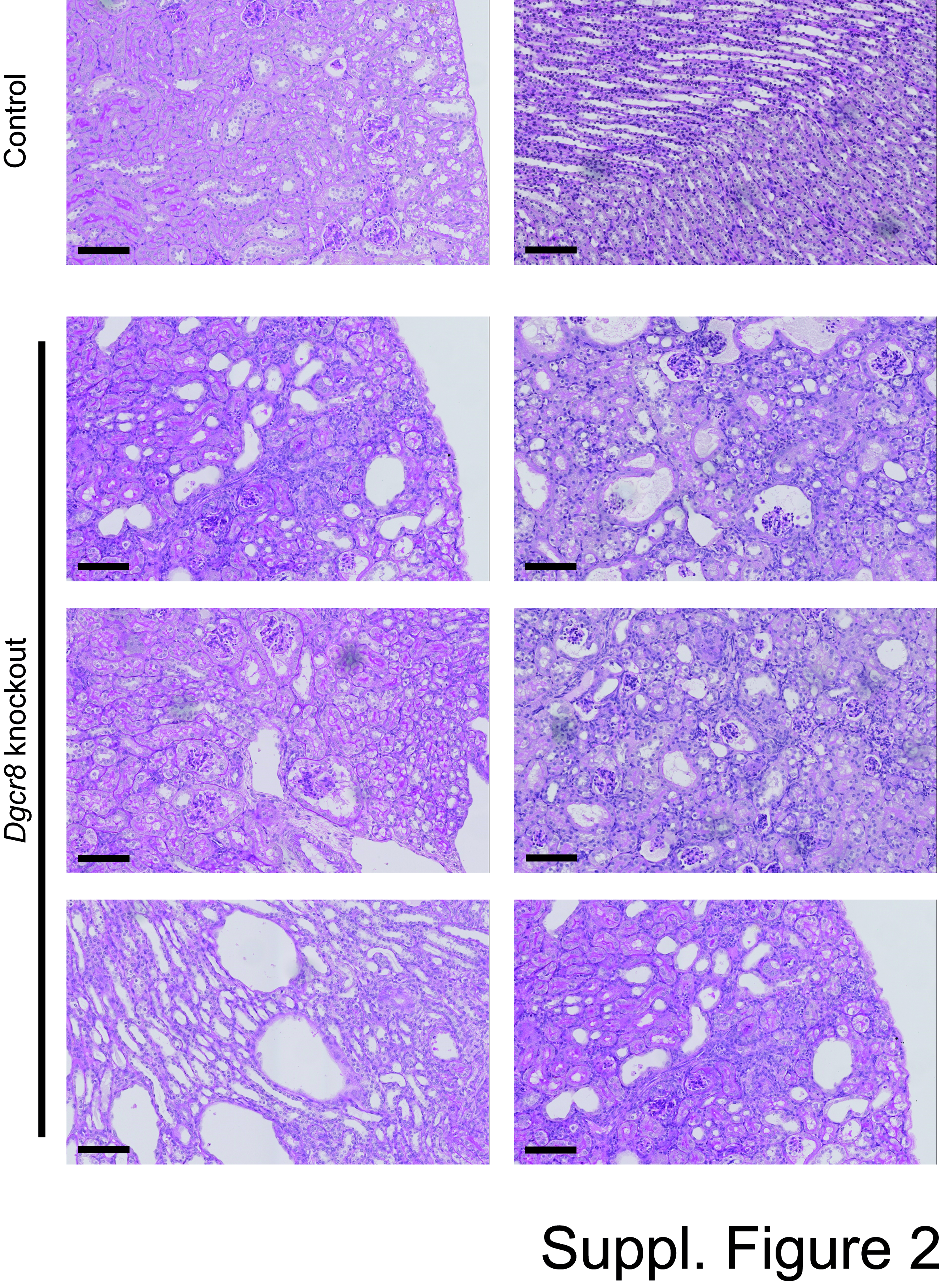

Supplement: Supplementary file 2 — 10.1186/s12867-016-0064-x Additional histological images of Dgcr8 knockout kidneys. PAS staining of the kidneys of Dgcr8 knockout mice shows cysts in the collecting duct, distal and proximal tubulus system, glomerular cysts with activated Bowman epithelium and a reduction in kidney parenchym (bar = 100 µm). [file 12867_2016_64_MOESM2_ESM.tif]

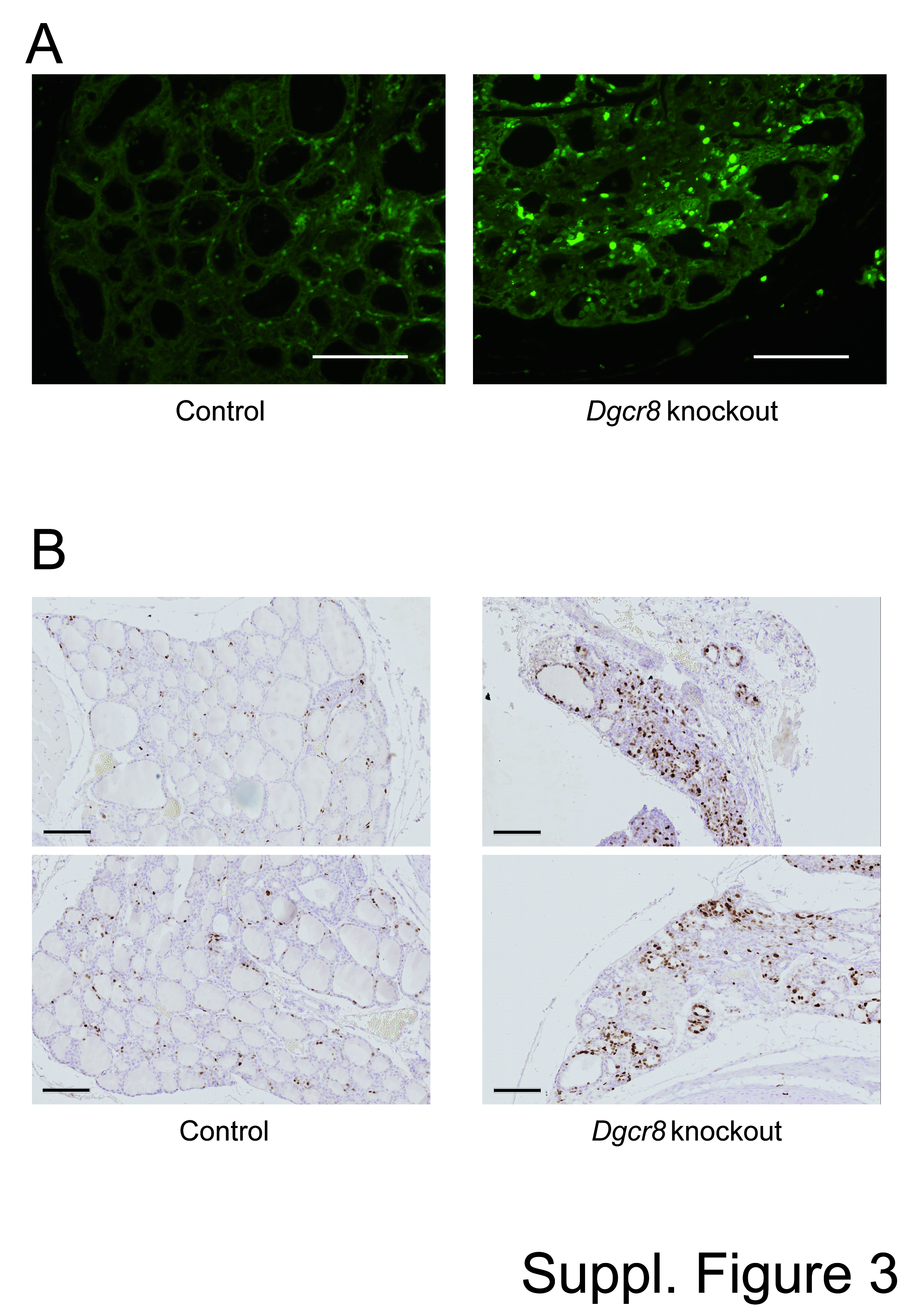

Supplement: Supplementary file 3 — 10.1186/s12867-016-0064-x Increased levels of apoptosis and proliferation in thyroid glands of Dgcr8-Pax8Cre knockout mice. A An increase in apoptotic cells in the thyroid gland of Dgcr8 knockout animals were detected by TUNEL assays (bar = 100 µm). B Similar to the kidney, there is a marked increase in proliferation as detected by Ki-67 stainings in the thyroid gland of Dgcr8 knockout mice (bar = 100 µm). [file 12867_2016_64_MOESM3_ESM.tif]
